# Quantitative Analysis of Cone Photoreceptor Distribution and Its Relationship with Axial Length, Age, and Early Age-Related Macular Degeneration

**DOI:** 10.1371/journal.pone.0091873

**Published:** 2014-03-14

**Authors:** Ryo Obata, Yasuo Yanagi

**Affiliations:** Department of Ophthalmology, University of Tokyo School of Medicine, Bunkyo-ku, Tokyo, Japan; Dalhousie University, Canada

## Abstract

**Purpose:**

It has not been clarified whether early age-related macular degeneration (AMD) is associated with cone photoreceptor distribution. We used adaptive optics fundus camera to examine cone photoreceptors in the macular area of aged patients and quantitatively analyzed its relationship between the presence of early AMD and cone distribution.

**Methods:**

Sixty cases aged 50 or older were studied. The eyes were examined with funduscopy and spectral-domain optical coherence tomography to exclude the eyes with any abnormalities at two sites of measurement, 2° superior and 5° temporal to the fovea. High-resolution retinal images with cone photoreceptor mosaic were obtained with adaptive optics fundus camera (rtx1, Imagine Eyes, France). After adjusting for axial length, cone packing density was calculated and the relationship with age, axial length, or severity of early AMD based on the age-related eye disease study (AREDS) classification was analyzed.

**Results:**

Patient’s age ranged from 50 to 77, and axial length from 21.7 to 27.5 mm. Mean density in metric units and that in angular units were 24,900 cells/mm^2^, 2,170 cells/deg^2^ at 2° superior, and 18,500 cells/mm^2^, 1,570 cels/deg^2^ at 5° temporal, respectively. Axial length was significantly correlated with the density calculated in metric units, but not with that in angular units. Age was significantly correlated with the density both in metric and angular units at 2° superior. There was no significant difference in the density in metric and angular units between the eyes with AREDS category one and those with categories two or three.

**Conclusion:**

Axial length and age were significantly correlated with parafoveal cone photoreceptor distribution. The results do not support that early AMD might influence cone photoreceptor density in the area without drusen or pigment abnormalities.

## Introduction

Age-related macular degeneration (AMD) is a leading cause of blindness in developed countries. [1] AMD has two stages; i.e., early AMD and late AMD. Drusen and pigment abnormalities are the hallmarks of early AMD. [2], [3] They are usually recognized in focal areas, but the pathological investigation proved diffusely distributed membranous deposits on the basement membrane of the retinal pigment epithelium throughout the macula. [4]–[6] Early AMD is predisposed to late AMD, which is characterized by development of choroidal neovascularization or progressive retinochoroidal atrophy resulting in severe vision loss. Susceptible genes and environmental risk factors have been reported, [7] which suggest that RPE damage is critical in AMD pathogenesis.

Photoreceptor loss is also documented in early AMD, disorganization of rod photoreceptor has been well demonstrated both pathologically [8], [9] and physiologically. [10] Meanwhile, alteration in cone photoreceptors has not been fully understood. Some previous studies pathologically demonstrated that cone photoreceptors were disorganized at the fovea or parafovea in early AMD patients. [9], [11] Other studies reported that central visual field, [12] cone adaptation, [13] blue cone sensitivity, [14] focal ERG, [15] and multifocal ERG [16] showed impaired cone function even in the early stage of the disease. It has also been demonstrated pathologically that cone photoreceptor density was decreased in the parafovea of three eyes with early AMD. [5] However, another study reported the photoreceptor damage was confined to areas directly overlying drusen. [17].

High-resolution retinal images using adaptive optics (AO) has been introduced recently,[18]–[21] making it possible to analyze photoreceptor distribution in areas of interest in living normal [22]–[24] or affected eyes [25]–[29] non-invasively. Regarding the influence of AMD, a pilot study described slight disruption in the cone photoreceptor mosaic in early AMD. [30] However, large number of subjects and adjustment for potentially confounding factors such as eccentricity to the fovea, axial length, or age are essential to clarify the influence of AMD on cone photoreceptor distribution.

Here we used AO fundus camera to examine cone photoreceptor distribution in the macular area of a relatively large number of aged patients and quantitatively analyzed the relationship between cone photoreceptor distribution and axial length, age, or the presence of early AMD.

## Materials and Methods

This observational case series study was approved by the institutional review board of University of Tokyo Graduate School of Medicine. Written consent was given by the patients for their information to be stored in the hospital and used for research. The study adhered to the tenets of the Declaration of Helsinki.

### Patients

Sixty-nine patients (37 men and 32 women; mean age 65.0 years, range 50–80 years) who visited Macular Clinic, University of Tokyo Hospital between September 2012 and October 2012 with unilaterally affected macular diseases were included. The unaffected eye was used for study. If the patients had any ocular diseases other than early AMD in the unaffected eye or the best-corrected decimal visual acuity (BCVA) in the unaffected eye was worse than 0.8, they were excluded from the study.

### Examination

Each patient underwent complete examination, including axial length measurement, anterior segment and fundus examination by slit-lamp biomicroscopy after pupil dialation. Fundus autofluorescence images were also acquired with HRAII (Heidelberg Engineering, Heidelberg, Germany), if possible. Axial length was measured with IOL master (Carl Zeiss Meditec, Jena, Germany). The study eye was classified into AMD category 1, 2, or 3 according to the criteria reported by age-related eye disease study group [31], [32] based on the fundoscopic findings within two disc diameters of the center of the macula. Briefly, category 1 included eyes with no or small (<63 μm) drusen, category 2 included eyes with intermediate (≥63, <125 μm) drusen or pigment abnormalities, and category 3 with large (≥125 μm) drusen.

**Figure 1:**
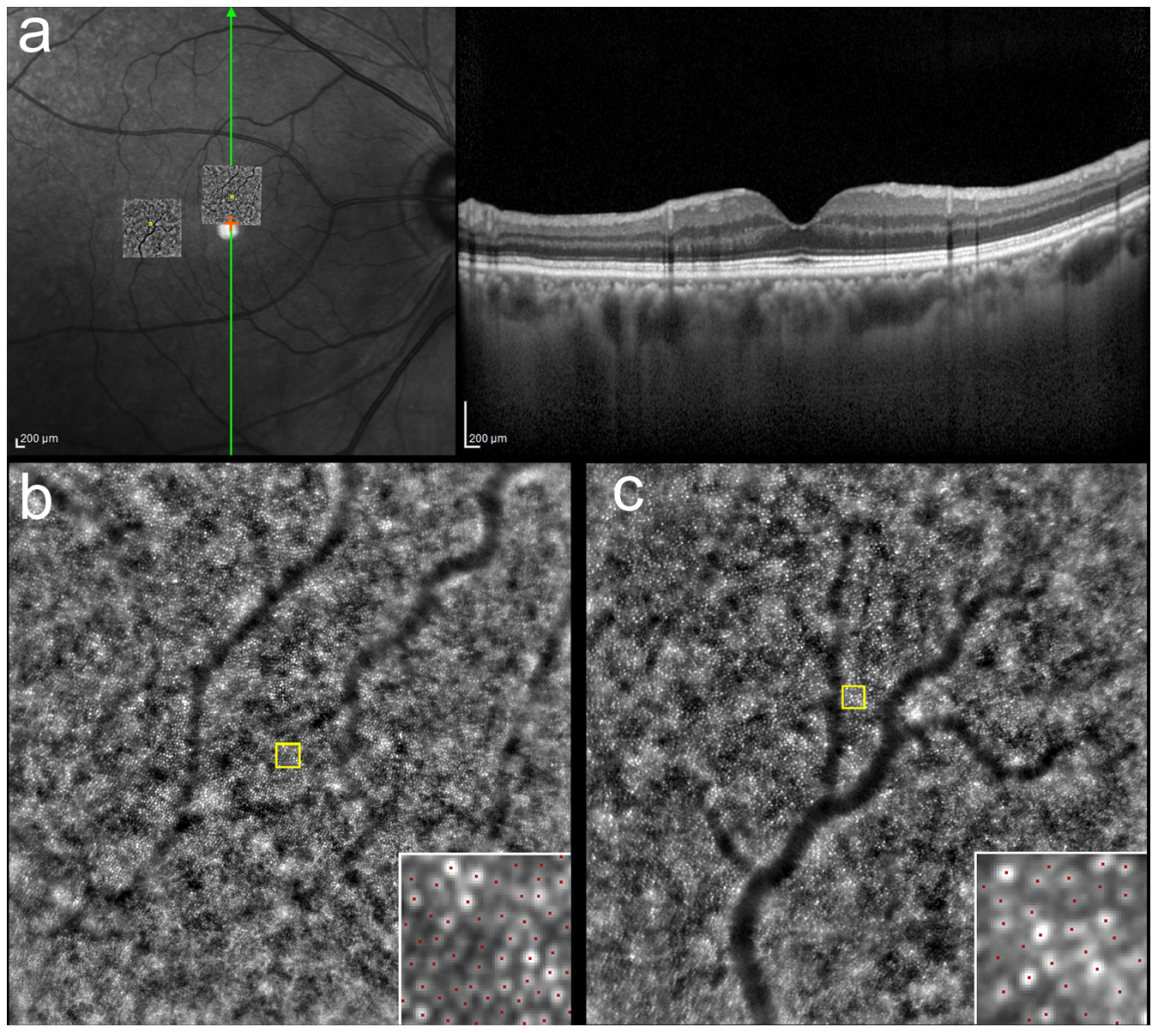
Identification of the sites for measurement in the images taken by adaptive optics fundus camera. Identification and measurement of cone distribution at 2° superior and 5° temporal to the fovea. Figures of a representative case are shown. Horizontal and vertical SD-OCT scan images centered on the fovea were obtained simultaneously with IR images using Spectralis (a). After the site corresponding to the fovea was identified on the IR image (orange cross in a) by referring to the OCT images, the sites at 2° superior and 5° temporal to the fovea on the IR image were located (yellow squares in a). Processed images from the adaptive optics (AO) fundus camera were overlaid with IR images by referring to retinal vessels in order to identify the sites of interest on AO images. A 60 pixel by 60 pixel square was placed at these sites (yellow square in b and c). Cone mosaic within the square was identified (red dots in the insets of 1b and 1c) and its distribution was assessed.

Spectral-domain optical coherence tomography (SD-OCT) images were obtained with SpectralisOCT (Heidelberg Engineering, Heidelberg, Germany). Thirty-degree horizontally or vertically scanned images centered on the fovea were taken. Using eye-tracking system, at most 100 tomographs captured at the same location were overlaid to decrease random speckle noise. Infrared (IR, 815 nm) reflectance images 30° by 30° were simultaneously obtained with OCT scan (**Figure 1**). By referring to the OCT images, the center of the fovea was located on the IR image. Then the IR image was imported into an open-source imaging program (GIMP, version 2.8.2). The points at 2° superior and 5° temporal to the fovea were located on the IR image, using the corresponding distance in pixel units calculated by dividing the pixel length of the whole IR image (30°) by 15 or 6. After the sites at 2° superior and 5° temporal to the fovea were located, funduscopy and the OCT scan were reviewed. If any drusen or RPE disturbance were detected at any of these points, it was excluded from the analysis.

### Adaptive Optics Fundus Camera

High-resolution retinal images with cone photoreceptor mosaic were obtained with flood-illuminated adaptive optics fundus camera (rtx1, Imagine Eyes, Orsey, France).[33]–[36] The rtx1 has a resolution of 1.6 μm with a 4.2° by 4.2° imaging field of view and an illumination wavelength of 850 nm. Patients were instructed to gaze a built-in fixation target that could be moved within ±10° horizontally and ±8° vertically. After checking whether the patient was properly fixating, 40 images were acquired during approximately four seconds. These images are processed and overlaid to yield a 4° by 4° highly contrasted image.

### Measurement of Cone Distribution

The processed AO image was imported into the imaging program (GIMP). It was manually overlaid with the IR image using the functions of resizing, parallel translation, or rotation by referring to retinal vessels. Thereby the locations at 2° superior and 5° temporal to the fovea were identified on the AO images. Cone distribution was measured at each site firstly using the software provided by the manufacturer (AOdetect Ver. 0.1. Imagine Eyes). [35] A 60 pixel by 60 pixel square was placed at 2° superior and 5° temporal to the fovea. The area was chosen not to include defects. The size of the square in each image was also expressed in the metric unit after it was calibrated with the axial length according to the formula by Bennett et al. [37]. In eyes with axial length of 24 mm, 60 pixel of the image corresponds approximately to 50 μm. Within the square, cone photoreceptor density and spacing were automatically calculated by cells/mm^2^ and by μm. Furthermore, Voronoi diagram was automatically constructed from each cone mosaic to calculate the proportion of hexagonal Voronoi domains, that indicates regularity of cone packing arrangement. [28], [38], [39] We also calculated cone angular density (cells/deg^2^). [23], [24] It was provided by dividing the absolute number of cones (cells) within this square by the area of the square (deg^2^). The area of the square (deg^2^) was obtained by multiplying the area of the AO image (equal to 16 deg^2^) by the proportion of the area of the square (pixel^2^) to that of the whole AO image (equal to 140,625 pixel^2^).

In all images, automatically detected cones were inspected and modified manually by two independent observers without knowledge of the backgrounds or the fundus image of each case. The inter-observer variability was calculated as the absolute difference in cone numbers between observers divided by the average cone number. If the variability was less than 5%, the average cone number was used as the final count to calculate the cone density. When the variability was 5% or more, two observers re-examined the image together and performed a third count as the final one.

### Statistical Analysis

Student t test was used for comparison of the background characteristics between eyes with AMD category 1 and those with AMD categories 2 or 3. Relationship between parameters of cone distribution and axial length, age, and AMD category was analyzed by multiple linear regression analysis. P value less than 0.05 was considered to be statistically significant. The software package JMP (SAS Institute, Cary, NC) was used for the analyses.

## Results

### Patient Demography

Of all 69 enrolled patients, nine patients were excluded because of blurred images which resulted from media opacity such as cataract or dry eye. Of the remaining 60 patients, the site at 2° superior to the fovea in 9 patients and that at 5° temporal in 12 patients were excluded from the measurement since drusen or RPE abnormality was detected by funduscopy or OCT scan. Therefore, the data at 2° superior of 51 eyes and that at 5° temporal of 48 eyes in 60 patients were used for analysis. The demography of the 60 patients was shown in **Table 1**. Mean age was 64.2 (range: 50–77). Thirty-two (53%) patients were unilaterally affected by neovascular AMD, and 28 (47%) by other diseases such as branch retinal vein occlusion (16 eyes), central retinal vein occlusion (5 eyes), idiopathic epiretinal membrane (4 eyes), and idiopathic full-thickness macular hole (3 eyes). Best-corrected decimal visual acuity ranged 0.8 to 1.2 (median, 1.0). Thirty (50%), 18 (30%), and 12 (20%) eyes were classified as AMD category 1, 2, and 3, respectively. Sex predominance, age, axial length, and proportion of neovascular AMD in the contralateral eye were not significantly different between the eyes in AMD category 1 and those in categories 2 or 3.

**Table 1:** Patient demographics.

	Total	AMD category	AMD categories
		1	2 or 3
No. of eyes	60	30	30
Male (%)	31 (52)	15 (50)	16 (53)
Mean axial length (mm)	23.9	24	23.8
[95% CI]	[23.6∶24.2]	[23.6∶24.5]	[23.2∶24.3]
(range)	(21.7–27.5)	(22.3–26.5)	(21.7–27.5)
Mean age	64.2	63.6	64.9
[95% CI]	[62.5∶65.9]	[60.9∶66.3]	[62.7∶67.0]
(range)	(50–77)	(50–77)	(53–77)
Neovascular AMD	32 (53)	14 (47)	18 (60)
in the affected eye (%)			

There was no statistically significant difference in background characteristics between eyes with AMD category 1 and those with categories 2 or 3.

### Parameters of Cone Distribution

Out of 60 patients, parameters of cone distribution at 2° in 51 patients and those at 5° in 48 patients were measured. Mean cone density in metric units and angular units, after manual modification were 24,900±3,400 cells/mm^2^, 2,170±400 cells/deg^2^, respectively, at 2° superior, and 18,500±2,600 cells/mm^2^, 1,570±140 cells/deg^2^, respectively, at 5° temporal(**Table 2**). Cone spacing in metric and angular units, and proportion of hexagonal Voronoi domains were automatically calculated and shown in Table S1. During manual modification, the inter-observer variability were 1.4±3.5% and 0.4±5.1% (mean ± SD) at 2° and 5°, respectively.

**Table 2:** Cone photoreceptor density in metric and angular units at 2° and 5° to the fovea.

	2° superior	5° temporal
Cone density (mean±SD)		
(cells/mm2)	24,900±3,400	18,500±2,600
[95%CI]	[24,000∶25,900]	[17,700∶19,200]
(cells/deg2)	2,170±400	1,570±140
[95%CI]	[2,060∶2,290]	[1,530∶1,610]

It was firstly automatically counted and then manually edited.

### Correlation with Axial Length, Age, and AMD Category

**Figure 2:**
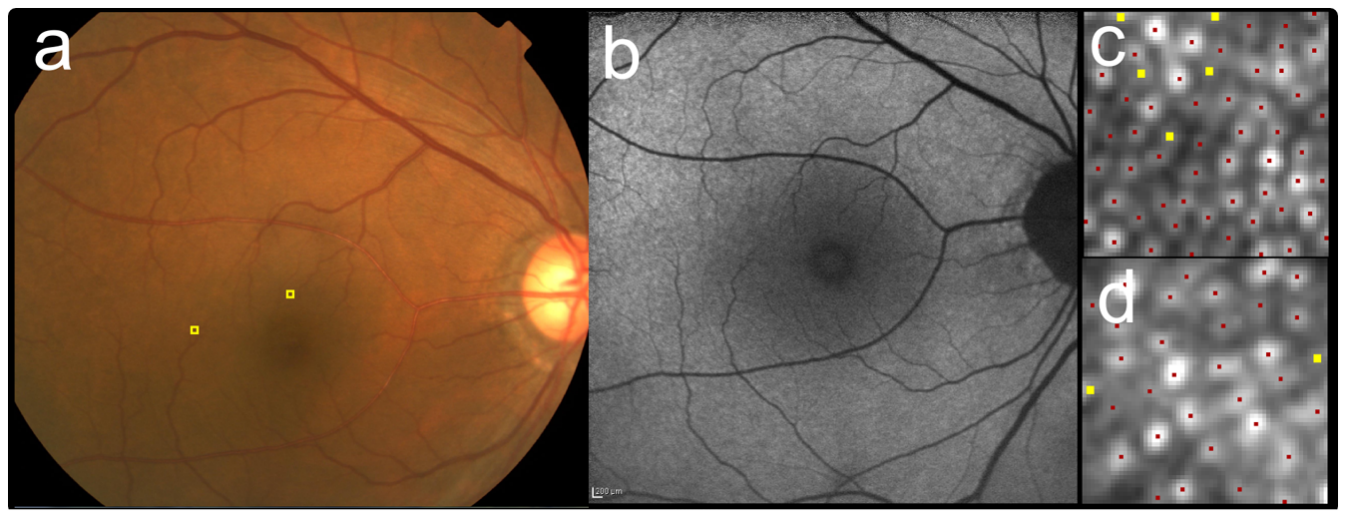
Cone mosaic images of the case with AREDS category 1 (no drusen). The right eye of 69-year-old male (the same eye as Figure 1) as a representative case with AREDS category 1 (no drusen). The fundus photo (**a**) did not show any sign of drusen or pigmentary abnormalities. The fundus autofluorescence (FAF) (**b**) was also unremarkable. After AO image was taken, the 60 pixel by 60 pixel square image was cropped at 2° superior (**c**) and 5° temporal (**d**) to the fovea (also shown as yellow squares in **a**). Cone mosaic was identified automatically at first (red dots in **c** and **d**), then added (yellow dots) in manual modification. Cone density were 25,500 cells/mm^2^ (2,340 cells/deg^2^) at 2° and 14,030 cells/mm^2^ (1,290 cells/deg^2^) at 5°.

Multiple linear regression coefficients analyzing effect of patient’s demography on cone densities are shown in **Table 3** and **Table 4**. Cone density in metric units was significantly correlated negatively both with axial length and age, but not with AMD category at 2° (**Table 3**) and with axial length but not with AMD category at 5° (**Table 4**). In contrast, cone density in angular units was significantly correlated only with age at 2° (**Table 3**). Additionally, cone density in metric and angular units were not significantly different between the patients with neovascular AMD and those with other diseases in the affected eye (data not shown). Representative cases with various AMD categories are shown in **Figure 2** **,** **3** **, and** **4**.

**Figure 3:**
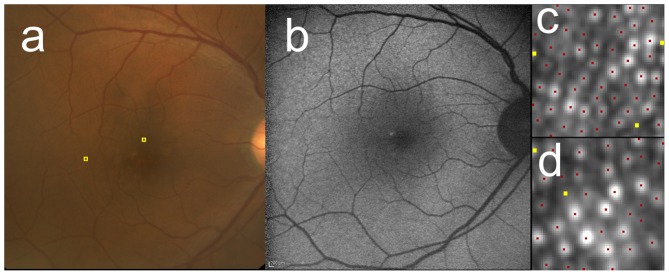
Cone mosaic images of the case with AREDS category 2 (pigmentary abnormality). The right eye of 59-year-old male with AREDS category 2. The fundus photo (**a**) showed hypopigmentation temporal to the fovea. FAF (**b**) showed irregular hyper- and hypofluorescence corresponding to the area. After AO image was taken, the 60 pixel by 60 pixel square image was cropped at 2° superior (**c**) and 5° temporal (**d**) to the fovea (also shown as yellow squares in **a**). Cone mosaic was identified automatically at first (red dots in **c** and **d**), then added (yellow dots) in manual modification. Cone density were 22,600 cells/mm^2^ (2,190 cells/deg^2^) at 2° and 14,900 cells/mm^2^ (1,450 cells/deg^2^) at 5°.

**Figure 4:**
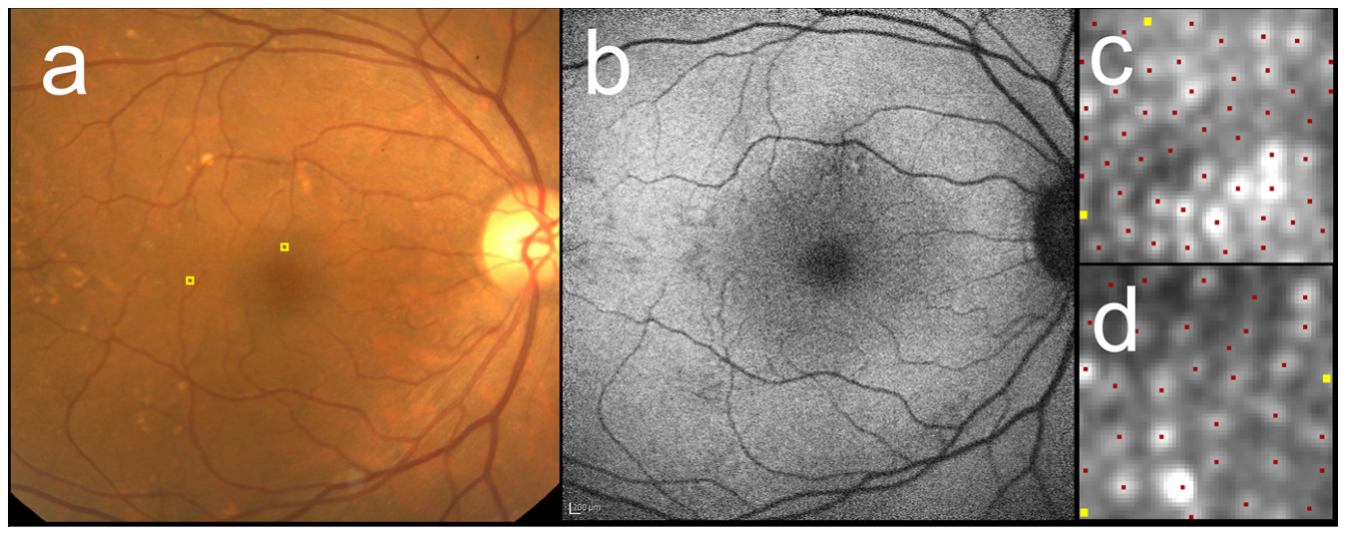
Cone mosaic images of the case with AREDS category 3 (large drusen). The right eye of 68-year-old female with AREDS category 3. The fundus photo (**a**) showed large drusen superior and temporal to the fovea. FAF (**b**) revealed hyper- and hypopigmentation corresponding to the drusen. After AO image was taken, the 60 pixel by 60 pixel square image was cropped at 2° superior (**c**) and 5° temporal (**d**) to the fovea (also shown as yellow squares in **a**). Cone mosaic was identified automatically at first (red dots in **c** and **d**), then added (yellow dots) in manual modification. Cone density were 25,700 cells/mm^2^ (1,990 cells/deg^2^) at 2° and 14,900 cells/mm^2^ (1,450 cells/deg^2^) at 5°.

**Table 3:** Multiple linear regression coefficients analyzing effects of demographic valuables on cone photoreceptor distribution at 2°.

	Retinal	Angular
	density	density
(Constant)		
P	<0.0001	0.022
b coefficient	67,500	3,160
[95%CI]	[45,400∶89,600]	[480∶5,850]
Axial length		
P	0.0002	0.56
b coefficient	−1,390	
[95%CI]	[−2,090:−683]	
Age		
P	0.042	0.0054
b coefficient	−147	−24.8
[95%CI]	[−287:−6]	[−41.9:−7.7]
AMD category (1 or 2–3)		
P	0.96	0.44
b coefficient		
[95%CI]		

Coefficients that were not statistically significant are not shown.

**Table 4:** Multiple linear regression coefficients analyzing effects of demographic valuables on cone photoreceptor distribution at 5°.

	Retinal	Angular
	density	density
(Constant)		
P	<0.0001	0.0009
b coefficient	56,800	1,840
[95%CI]	[43,000∶70,600]	[800∶2,880]
Axial length		
P	<0.0001	0.77
b coefficient	−1,510	
[95%CI]	[−1,970:−1,060]	
Age		
P	0.43	0.47
b coefficient		
[95%CI]		
AMD category (1 or 2–3)		
P	0.72	0.95
b coefficient		
[95%CI]		

Coefficients that were not statistically significant are not shown.

## Discussion

In the present study, we used adaptive optics (AO) fundus camera to examine cone photoreceptor distribution in the macular area of aged patients and, quantitatively analyzed its relationship with age, axial length, and early age-related macular degeneration (AMD). Since previous reports suggested that cone distribution might vary according to eccentricity to the fovea, axial length, or age, quantitative analyses investigating any difference between patients and controls should take these confounding factors into consideration. Therefore we examined cone distribution at the specific eccentricities with adjustment for these confounding factors.

As previously discussed, acquisition of fundus images of aged patients is technically challenging. [30] The difficulty derives from various factors such as unstable fixation or opaque media. In the present study, we selected patients with good visual acuity to address this problem. Although part of the examined eyes was still excluded from the study because of blurred images by cataract or dry eye, we were able to obtain the AO images from a number of the aged patients with good to excellent repeatability.

In cone counting, automatic counting software is useful and will be essential when large quantity of data is to be analyzed. However, currently, manually modified count is more reliable. [38], [40], [41] In the present study automatic counting was manually edited by two observers. Inter-observer differences were 0.4 to 1.4%, and in the cases with variability 5% or higher, recount was performed to enhance the reliability of manual modification.

Axial length negatively influenced on cone density calculated in metric units both at 2° and 5°, while it did not if calculated in angular units. As previously discussed,[22]–[24], [39] axial length is a major variable to be taken care of in calculating cone distribution outside the fovea. There may be two reasons; Firstly, a particular angular eccentricity corresponds to different distances from the fovea in metric units with different axial lengths. For example, 2° and 5° eccentricity corresponds approximately to 0.53 to 0.64 and 1.3 to 1.6 mm with axial length of 22 to 26 mm, respectively. Secondly, as axial length increases, retinal size in metric units of a particular angular area increases, leading cone density to less value when calculated in metric units. Therefore we measured at the same eccentricities from the fovea described in angular units and calculated cone density both in metric units and angular units. In a previous report [22] studying 19 normal subjects aged 20 to 52 with AO imaging, eyes with moderate to high myopia showed significantly longer cone spacing in metric units than those with normal to low myopia. Other studies [23], [24] investigated cone distribution in young healthy eyes and reported that cone density in metric units negatively correlated with axial length while angular density shows no significant correlation. The results of the present study indicate that this relationship also applies to aged patients. Recently another study showed that axial length was significantly correlated with cone density in metric units at 0.5 mm eccentricity from the fovea, while not at 1.0 mm and 1.5 mm eccentricities. 1.5 mm eccentricity corresponds to approximately 5°. [39] Discrepancy with the result of the present study may be partly because, as discussed in the previous report, [24] when eccentricity was set in metric units, the eccentricity calculated in angular units becomes narrower with longer axial length, and it may tend to attenuate the negative relationship between cone density in metric units and axial length.

Additionally age was negatively correlated with cone density after adjustment for axial length at 2 degrees eccentric to the fovea. Decrease in cone function with age was reported in a physiological study. [42] Transfer of metabolic products across the RPE layer is impaired with the accumulation of age-related deposits in Bruch’s membrane and retinal pigment epithelium, and the resultant insufficiency of the nutrients and ischemia is indicated to lead to dysfunction of cone photoreceptors. [8] Histologically cone photoreceptor decreases at the parafoveal lesion [43] and appreciable number of nuclei was displaced from the outer nuclear layer to the photoreceptor layer over the age of 40, causing disarray of photoreceptor inner or outer segment. [44] Inner segments also showed deposition of lipofuscin with aging [45], [46] and outer segment revealed accumulation of amyloid beta, [47] indicating disorganization within inner or outer segments with aging. Since AO imaging detects the reflected light guided through the inner and outer segment, [20] the loss, disarray, and/or disorganization with aging should influence in the number of cones detected by AO imaging. Indeed, age-related decline in the cone density in the parafoveal area was previously suggested. [48], [49] Another recent study also reported that cone density in the parafovea showed a trend towards negative correlation to age. [39] The result in the present study, with large number of patients, was compatible with these report.

Several reports investigated cone density in normal subjects histologically or using AO imaging. They showed that cone density rapidly decreases with increasing eccentricity, with the value between approximately 35,000 and 15,000 cells/mm^2^ at 0.5 mm to 1.0 mm to the fovea. [27], [39], [48] In contrast, cone density decreases more slowly at the more eccentric area, with the value from approximately 19,000 to 11,000 cells/mm^2^ at 1.0 mm to 1.9 mm to the fovea. [27], [39], [48] However, these values were not fully adjusted for axial length, the unit used for indicating eccentricity, and horizontal or vertical meridian, all of which were reported to influence cone density. [39], [48], [50] Even after adjusting them, considerable individual variation have been observed. [23], [48], [51] Therefore it is difficult to directly compare the cone density from different studies. However, it should be noted that the mean cone density at 2° in the present study seems slightly lower than a previous report [48] in that 10 normal eyes aged 50 to 65 showed 29,400 cells/mm^2^ at 0.54 mm and 23,200 cells/mm^2^ at 0.72 mm to the fovea. Since the slight decrease was observed only at 2° but not at 5°,it is unlikely to be explained by impaired image quality from media opacity or increased aberration in the aged patients. The previous study [48] reported that, comparing normal subjects aged 50 to 65 with those aged 22 to 35, there was significant decrease in cone density only within 0.5 mm to the fovea. Although the mechanism remains unclear, the current findings, together with previous studies, seems to suggest that as the subjects become older, cone density decreases at the parafovea.

In the present study, cone distribution was not significantly different between eyes with low and high severity of early AMD or between patients affected with neovascular AMD and other macular diseases in the contralateral eye. Although cone dysfunction has been demonstrated in eyes with early AMD, alteration in cone distribution in the area without drusen or pigmentary abnormalities has not been clarified. Therefore, we examined the region where drusen or pigmentary abnormalities were not detected. The results of this study do not support that severity of early AMD might be associated with cone photoreceptor distribution in the area without drusen or pigment abnormalities. For future analysis, it will be of much interest to analyze the cone distribution overlying drusen or pigmentary abnormalities by taking images with sufficient quality and adjusting several aforementioned confounding factors.

However, there are some limitations in the study such as retrospective nature of the study design. Additionally the sites of measurement were restricted to small area. Nevertheless, these results indicate that AO imaging technique can be used to evaluate cone distribution of aged patients with good visual acuity and transparent media, and might contribute to understanding in the relationship between cone distribution and confounding factors in aged patients or AMD.

In conclusion, we used AO fundus camera to examine cone photoreceptor distribution in the macular area of aged patients and quantitatively analyzed its relationship with age, axial length, and early age-related macular degeneration (AMD). Axial length and age showed significant correlation with parafoveal cone photoreceptor distribution. Severity of early AMD may not be associated with cone distribution in the area without apparent drusen or pigment abnormalities. AO imaging can be used to assess cone photoreceptor distribution of aged patients and might be helpful to clarify the relationship between cone photoreceptor distribution, aging, and AMD.

## Supporting Information

Table S1 Cone photoreceptor spacing in metric and angular units and hexagonal Voronoi domains at 2° and 5° to the fovea. It was automatically measured by the software created by manufacturer.
(RTF)
