# Supplementary material for: Quantitative Analysis of Cone Photoreceptor Distribution and Its Relationship with Axial Length, Age, and Early Age-Related Macular Degeneration
Source: PLoS One. 2014 Mar 14;9(3):e91873. doi: 10.1371/journal.pone.0091873 (PMC3954804; doi:10.1371/journal.pone.0091873)
Supplement: Table S1 — Cone photoreceptor spacing in metric and angular units and hexagonal Voronoi domains at 2° and 5° to the fovea. It was automatically measured by the software created by manufacturer. (RTF) [file pone.0091873.s001.rtf]

SUPPORTING INFORMATION
Table S1. Cone photoreceptor spacing in metric and angular units and hexagonal Voronoi domains at 2° and 5° to the fovea. It was automatically measured by the software created by manufacturer.

	2° superior	5° temporal	
Cone spacing (mean±SD)			
 (ìm)	7.57±0.69	9.09±0.78	
 [95%CI]	[7.38:7.77]	[8.86:9.31]	
 (arcmin) 	1.56±0.13	1.87±0.13	
 [95%CI] 	[1.52:1.59]	[1.83:1.91]	
			
Hexagonal Voronoi domains			
(%)	49.2	44.1	
 [95%CI]	[46.2:52.1]	[40.9:47.2]	
